# Multifocal Giant Granular Cell Tumors of the Alimentary Canal With Concurrent Eosinophilic Gastrointestinal Disease

**DOI:** 10.14309/crj.0000000000002261

**Published:** 2026-08-03

**Authors:** Anil Mathew Philip, Daryl Forney, Lina James George, Saksham Kohli, Victoria Alagiozian-Angelova, Anas Almoghrabi

**Affiliations:** 1 Department of Internal Medicine, John H. Stroger Hospital of Cook County, Chicago, IL; 2 Chicago College of Osteopathic Medicine, Midwestern University, Downers Grove, IL; 3 Department of Pathology and Laboratory Medicine, John H. Stroger Hospital of Cook County, Chicago, IL; 4 Department of Gastroenterology and Hepatology, John H. Stroger Hospital of Cook County, Chicago, IL

**Keywords:** granular cell tumor, eosinophilic esophagitis, eosinophilic gastroenteritis, eosinophilic colitis, neuroendocrine tumor, eosinophilic gastrointestinal disease

## Abstract

Granular cell tumors (GCTs) are rare, typically benign Schwann cell neoplasms; within the gastrointestinal (GI) tract, the esophagus is the most frequent site. Eosinophilic gastrointestinal diseases are chronic immune-mediated conditions encompassing eosinophilic esophagitis, gastroenteritis, and colitis. We describe a 49-year-old man with prior testicular seminoma, follicular thyroid carcinoma, and cutaneous GCTs who developed multifocal esophageal and rectal GCTs alongside eosinophilic esophagitis, esophagitis gastroenteritis, and esophagitis colitis, plus an incidental sigmoid neuroendocrine tumor (WHO grade 2). To our knowledge, this is the first reported case of concurrent multisegmental eosinophilic gastrointestinal diseases, multifocal GCTs, and a neuroendocrine tumors (NET), highlighting the need for vigilant endoscopic surveillance.

## INTRODUCTION

Granular cell tumors (GCTs) are uncommon Schwann cell neoplasms that most frequently involve the esophagus within the gastrointestinal tract, where they account for approximately 1% of benign esophageal tumors.^1,2^ Prior studies have demonstrated a significant association between esophageal GCTs and eosinophilic esophagitis (EoE), with a reported co-occurrence odds ratio of 10.43.^3^

Eosinophilic gastrointestinal diseases (EGIDs) comprise eosinophilic esophagitis, gastroenteritis, and colitis and are characterized by eosinophilic infiltration of the gastrointestinal tract in the absence of secondary causes.^4,5^ However, the coexistence of multifocal GCTs involving multiple gastrointestinal sites with multisegment EGIDs has rarely been described. We report a patient with multifocal gastrointestinal GCTs; eosinophilic involvement of the esophagus, small bowel, and colon; and a concurrent neuroendocrine tumor, highlighting an unusual clinical association.

## CASE REPORT

A 49-year-old man with a history of testicular seminoma (status post orchiectomy and radiation), follicular thyroid carcinoma, chronic obstructive pulmonary disease, and multiple cutaneous GCTs excised in 2008 presented in 2021 with epigastric pain, diarrhea, weight loss, and dysphagia. Upper endoscopy demonstrated a 1.7 cm gastric nodule and esophageal eosinophilia of 50–70 eosinophils/high-power field (hpf), establishing a diagnosis of eosinophilic esophagitis (EoE) (Figure 5). Peripheral eosinophilia (700–800/µL) and elevated IgE (485 kU/L) were present. Secondary causes of gastrointestinal eosinophilia were excluded, including infectious etiologies (negative stool cultures, ova and parasites, Giardia, Strongyloides, and Clostridioides difficile testing), and there was no evidence of inflammatory bowel disease or other alternative causes. Proton-pump inhibitor therapy and budesonide were initiated.

Eight weeks later, repeat endoscopy revealed multifocal esophageal GCTs and a persistent gastric nodule (Figure 2). Concurrent colonoscopy identified submucosal lesions in the cecum, sigmoid colon, and rectum (Figure 3). Histopathology demonstrated a rectal GCT (CD68^+^, S100+) (Figure 4), a well-differentiated sigmoid neuroendocrine tumor (WHO grade 2, Ki-67 6%), and eosinophilic infiltration of the duodenum, ileum, and colon, consistent with eosinophilic gastroenteritis and eosinophilic colitis. Together, these findings established multisegment EGID involving the esophagus, small bowel, and colon. The rectal GCT and malignant neurendocrine tumors underwent complete resection via transanal minimally invasive surgery, with clear margins.

In July 2022, the patient developed recurrent diarrhea and progressive dysphagia. Endoscopy demonstrated multiple large esophageal submucosal nodules and a gastroesophageal junction stricture requiring balloon dilation. Biopsies again showed active EoE (≥50 eosinophils/hpf). Following transanal minimally invasive surgery, pathology from flexible sigmoidoscopy demonstrated chronic active proctitis.

**Figure 1.**
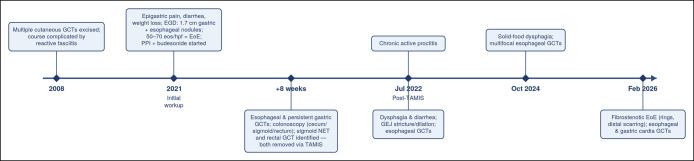
Clinical timeline.

In October 2024, repeat endoscopy for recurrent solid-food dysphagia demonstrated persistent multifocal esophageal GCTs (10–25 mm) and ongoing EoE. By February 2026, the patient had developed fibrostenotic EoE with diffuse esophageal rings and distal esophageal scarring. Additional GCTs were identified at 25 and 30 cm within the esophagus and within a gastric cardia lesion. The patient remains on budesonide and twice-daily proton-pump inhibitor therapy with ongoing endoscopic surveillance. Timeline of manifestation of the disease is shown in Figure 1

## DISCUSSION

Our case is unique as the patient experienced co-occurrence of pan-alimentary EGIDs, multifocal GCTs across distinct GI sites, and the concomitant presence of a neuroendocrine tumor.

EoE is the most common EGID, while EoC is the rarest.^2^ EoE is a chronic allergic inflammatory disease diagnosed by endoscopy and biopsy demonstrating ≥15 eosinophils per hpf and presents with dysphagia and food impaction in adults, and vomiting, weight loss, and heartburn.^3^ The prevalence of EoE in the United States is approximately 142.5 cases per 100,000 people.^4^ EoE is a Th2-mediated inflammatory disease often described as “inside out,” in which allergen penetration through defects in the esophageal barrier, driven by alterations in proteins such as filaggrin and calpain 14, initiates the inflammatory cascade.^6,7^

Given the chronic and relapsing nature of EoE, understanding treatment strategies is crucial. Proton-pump inhibitors (PPIs) and swallowed topical steroids, such as budesonide or fluticasone, are common first-line therapies and are the only pharmacologic therapy to receive a strong recommendation in American Gastroenterological Association guidelines.^8,9^ For patients nonresponsive to PPIs, dupilumab, the first approved biologic, is recommended as a second-line option.^8,10^ Esophageal dilation is used for significant luminal narrowing, though it does not address inflammation.^8^ EoE progresses from an inflammatory phenotype to fibrostenotic disease, with late-stage complications including esophageal stricture, food impaction (present in 30%–45% of patients), malnutrition, and neoplastic associations.^6,8,11,12^ This fibrostenotic progression is evident in our patient, whose disease advanced from recurrent dysphagia requiring dilation to diffuse esophageal rings and distal esophageal scarring.

Beyond EoE, our patient had widespread multisystem eosinophilic involvement. EoGE is a rare, benign inflammatory condition characterized by eosinophilic infiltrates in the stomach and small intestine, with a female predominance in contrast to EoE.^13^ EoGE is frequently associated with other atopic disorders such as asthma, allergic rhinitis, and food intolerances, identified in 45%–63% of individuals.^13,14^

EoC is the least common EGID, characterized by eosinophilic infiltrates in the large intestine in the absence of secondary causes. It has a bimodal age distribution, presenting as self-limited bloody diarrhea in infants and a more chronic colitis in adults.^1^

The patient in our case developed multiple GCTs throughout the alimentary canal, including multiple lesions in the esophagus and rectum. GCTs are rare, typically benign neoplasms of Schwann cells (neuroectodermal origin), confirmed by immunohistochemical staining with S100.^15,16^ Current data suggest that the co-occurrence of eGCTs and EoE is associated with a statistically significant odds ratio of 10.43, hypothesized to reflect reactive neoplasia arising from areas of chronic inflammation or tissue injury.^11^ Riffle et al and Zheng et al described EoE with esophageal GCT, and Nojkov et al established the epidemiologic association, but none reported multifocal extraesophageal GCTs or a coexisting neuroendocrine tumors (NET).^2,13,17^ To our knowledge, no prior report describes concurrent multisegmental EGIDs, multifocal GCTs, and a NET.

Importantly, this report demonstrates association, not causation. The temporal and spatial overlap of chronic eosinophilic inflammation with neural and neuroendocrine neoplasia is hypothesis-generating rather than mechanistic and may be coincidental given the patient's broader tumor-predisposition phenotype.

The patient in this case presented an exceptionally rare clinical profile due to the multifocal nature of his GCTs across multiple systems such as skin, esophagus, gastric cardia, and rectum, alongside eosinophilic processes throughout the alimentary canal supporting diagnoses of EoE, EoGE, and EoC.^16^ The discovery of a concomitant neuroendocrine tumor in the sigmoid colon further adds to the complexity of this case. This case highlights the potential interplay between chronic eosinophilic inflammation and neoplastic processes, emphasizing the need to further investigate shared pathogenic mechanisms and their clinical implications.

**Figure 2.**
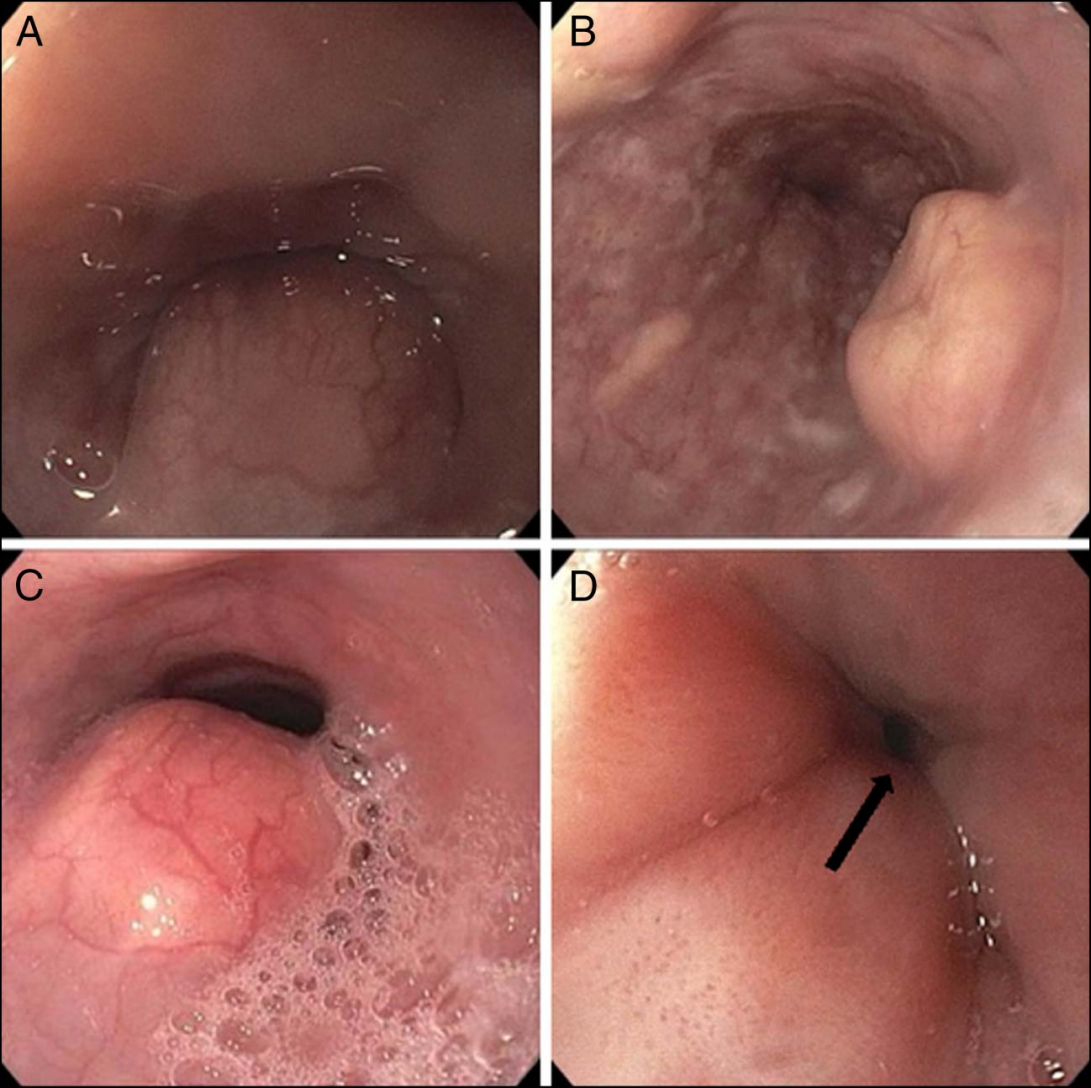
Upper endoscopic findings in a patient with multiple giant GCTs of the esophagus and subsequent development of a distal esophageal stricture. (A) A large, smooth-surfaced, pale submucosal nodule in the esophagus with overlying intact mucosa, representative of one of the GCT lesions. (B) A second, bulky submucosal mass with irregular, nodular surface texture and whitish discoloration, causing partial luminal narrowing, consistent with a giant GCT. (C) An additional sessile, erythematous submucosal lesion with prominent overlying vascularity, representing a third GCT at a separate esophageal level. The multiplicity of lesions raises consideration of a syndromic association. (D) A tight distal esophageal stricture attributed to underlying eosinophilic esophagitis, a coexisting diagnosis in this patient. The stricture is characterized by concentric luminal narrowing with loss of normal mucosal pliability, as shown with black arrowhead, reflecting the subepithelial fibrosis and remodeling that occur as sequelae of chronic eosinophilic inflammation. GCT, granular cell tumor.

**Figure 3.**
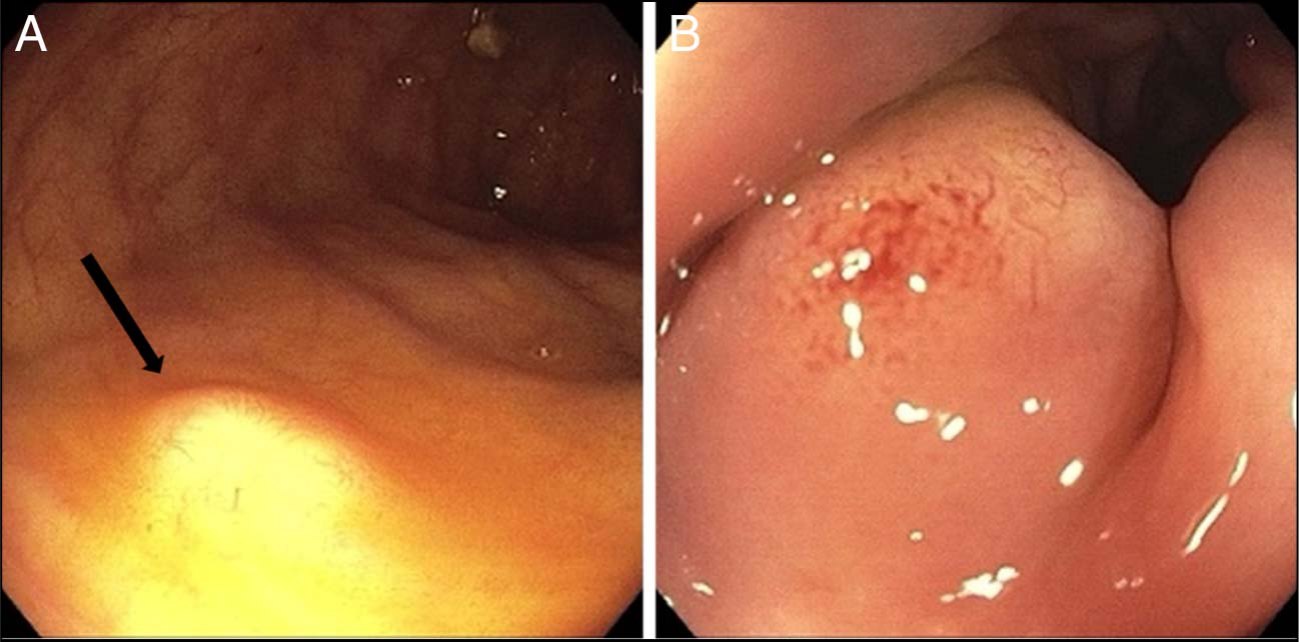
Colonoscopic appearance of GCTs at 2 anatomical sites. (A) A well-circumscribed, smooth-surfaced, sessile submucosal lesion in the cecum with overlying intact mucosa and characteristic yellowish-white discoloration, consistent with a granular cell tumor as shown with black arrowhead. (B) A sessile, dome-shaped submucosal nodule in the rectum with a central area of erythema and superficial mucosal irregularity. The endoscopic appearances at both sites are characteristic of GCT, with firm, pale submucosal nodules that indent but do not infiltrate the overlying mucosa. GCT, granular cell tumor.

**Figure 4.**
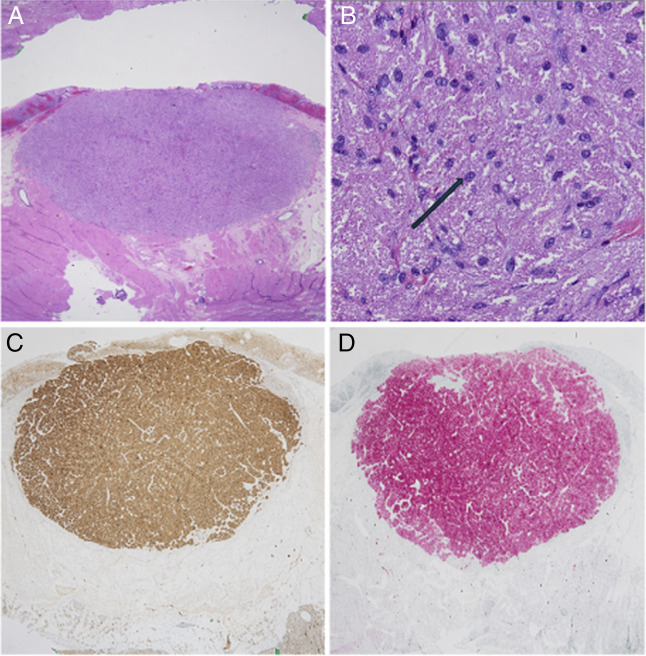
Histopathology and immunohistochemistry of GCT of the esophagus. (A) Low-power view (100×) demonstrating GCT involving squamous-columnar junction mucosa with associated surface epithelial denudation. (b) High-power (400×) view showing characteristic large polygonal cells with abundant eosinophilic granular cytoplasm and small, centrally located nuclei as shown with black arrowhead. (c) CD68 immunostaining showing diffuse cytoplasmic positivity consistent with lysosomal content of GCT. (d) S100 immunostaining demonstrating strong, diffuse positivity confirming neural/Schwann cell origin. The immunohistochemical profile (CD68^+^, S100+) supports the diagnosis of granular cell tumor. GCT, granular cell tumor.

**Figure 5.**
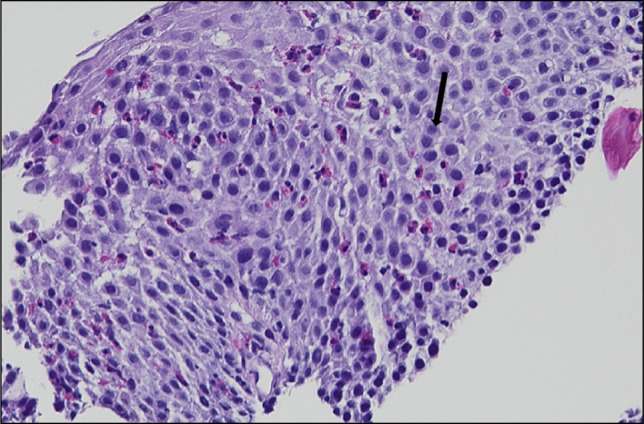
Esophageal biopsy (hematoxylin and eosin, high-power field; 400×) from the distal esophagus demonstrating marked intraepithelial eosinophilia. There is a diffuse and dense infiltration of eosinophils throughout the squamous epithelium, with peak eosinophil counts exceeding 60 eos/HPF as shown with black arrowhead, well above the diagnostic threshold of ≥15 eos/HPF for eosinophilic esophagitis. The eosinophils are distributed across all epithelial layers with associated spongiosis. These histopathological findings are consistent with a diagnosis of eosinophilic esophagitis. eos/HPF, eosinophils per high-power field.

## DISCLOSURES

Author contributions: AM Philip: substantial contributions to conception and design; drafting and revising the manuscript for important intellectual content; final approval of the version to be published; agreement to be accountable for all aspects of the work. D. Forney: substantial contributions to conception and design; drafting and revising the manuscript for important intellectual content; final approval of the version to be published; agreement to be accountable for all aspects of the work. LJ George: acquisition of clinical data; critical revision of the manuscript for important intellectual content; final approval of the version to be published; agreement to be accountable for all aspects of the work. S. Kohli: critical revision of the manuscript for important intellectual content; review and editing; final approval of the version to be published; agreement to be accountable for all aspects of the work. V. Alagiozian-Angelova: acquisition and interpretation of histopathological data; preparation of pathology images and descriptions; critical revision of the manuscript; final approval of the version to be published; agreement to be accountable for all aspects of the work. A. Almoghrabi: substantial contributions to conception and design; supervision; critical revision of the manuscript for important intellectual content; final approval of the version to be published; agreement to be accountable for all aspects of the work. All authors have reviewed and approved the final version of the manuscript. AM Philip is the article guarantor.

Financial disclosure: None to report.

Informed consent was obtained for this case report.

## ABBREVIATIONS:

EDIDs: Eosinophilic gastrointestinal diseases
EoC: Eosinophilic colitis
EoE: Eosinophilic esophagitis
EoGE: Eosinophilic gastroenteritis
GCT: Granular cell tumors
NET: Neuroendocrine tumor
PPI: Proton pump inhibitor
